# Prediction of Preterm Deliveries from EHG Signals Using Machine Learning

**DOI:** 10.1371/journal.pone.0077154

**Published:** 2013-10-28

**Authors:** Paul Fergus, Pauline Cheung, Abir Hussain, Dhiya Al-Jumeily, Chelsea Dobbins, Shamaila Iram

**Affiliations:** Applied Computing Research Group, Liverpool John Moores University, Liverpool, Merseyside, United Kingdom; New Jersey Institute of Technology, United States of America

## Abstract

There has been some improvement in the treatment of *preterm* infants, which has helped to increase their chance of survival. However, the rate of premature births is still globally increasing. As a result, this group of infants are most at risk of developing severe medical conditions that can affect the respiratory, gastrointestinal, immune, central nervous, auditory and visual systems. In extreme cases, this can also lead to long-term conditions, such as cerebral palsy, mental retardation, learning difficulties, including poor health and growth. In the US alone, the societal and economic cost of *preterm* births, in 2005, was estimated to be $26.2 billion, per annum. In the UK, this value was close to £2.95 billion, in 2009. Many believe that a better understanding of why *preterm* births occur, and a strategic focus on prevention, will help to improve the health of children and reduce healthcare costs. At present, most methods of *preterm* birth prediction are subjective. However, a strong body of evidence suggests the analysis of uterine electrical signals (Electrohysterography), could provide a viable way of diagnosing true labour and predict *preterm* deliveries. Most Electrohysterography studies focus on true labour detection during the final seven days, before labour. The challenge is to utilise Electrohysterography techniques to predict *preterm* delivery earlier in the pregnancy. This paper explores this idea further and presents a supervised machine learning approach that classifies *term* and *preterm* records, using an open source dataset containing 300 records (38 *preterm* and 262 *term*). The synthetic minority oversampling technique is used to oversample the minority preterm class, and cross validation techniques, are used to evaluate the dataset against other similar studies. Our approach shows an improvement on existing studies with 96% *sensitivity*, 90% *specificity*, and a 95% area under the curve value with 8% global error using the polynomial classifier.

## Introduction

*Preterm* birth, also known as premature birth or delivery, is described by the World Health Organisation (WHO) as the delivery of babies who are born, alive, before 37 weeks of gestation [1]. In contrast, *term* births are the live delivery of babies after 37 weeks, and before 42 weeks. According to the WHO, worldwide in 2010, *preterm* deliveries accounted for 1 in 10 births [1]. In 2009, in England and Wales, 7% of live births were also *preterm* (http://ons.gov.uk). *Preterm* birth has a significant adverse effect on the new born, including an increased risk of death and health defects. The severity of these effects increases the more premature the delivery is. Approximately, 50% of all perinatal deaths are caused by *preterm* delivery [2], with those surviving often suffering from afflictions, caused by the birth. These include impairments to hearing, vision, the lungs, the cardiovascular system and non-communicable diseases; up to, 40% of survivors of extreme *preterm* delivery can also develop chronic lung disease [3]. In other cases, survivors suffer with neuro-developmental or behavioural defects, including cerebral palsy, motor, learning and cognitive impairments. In addition, *preterm* births also have a detrimental effect on families, the economy, and society. In 2009, the overall cost to the public sector, in England and Wales, was estimated to be nearly £2.95 billion [4]. However, developing a better understanding of *preterm* deliveries can help to create preventative strategies and thus positively mitigate, or even eradicate, the effects that *preterm* deliveries have on babies, families, and society and healthcare services.

*Preterm* births can occur for three different reasons. According to [2], roughly one-third are medically indicated or induced; delivery is brought forward for the best interest of the mother or baby. Another third occurs because the membranes rupture, prior to labour, called Preterm Premature Rupture of Membranes (*PPROM*). Lastly, spontaneous contractions (termed *preterm* labour or *PTL*) can develop. However, there is still a great deal of uncertainty about the level of risk each factor presents, and whether they are causes or effects. Nevertheless, in [2] some of the causes of *preterm* labour, which may or may not end in *preterm* birth, have been discussed. These include infection, over-distension, burst blood vessels, surgical procedures, illnesses and congenital defects of the mother's uterus and cervical weakness. Further studies have also found other risk factors for *PTL*/*PPROM* [5], [6]. These include a previous *preterm* delivery (20%); last two births have been *preterm* (40%), and multiple births (twin pregnancy carries a 50% risk). Other health and lifestyle factors also include cervical and uterine abnormalities, recurrent antepartum haemorrhage, illnesses and infections, any invasive procedure or surgery, underweight or obese mothers, ethnicity, and social deprivation, long working hours/late nights, alcohol and drug use, and folic acid deficiency.

As well as investigating *preterm* deliveries, several studies have also explored *preterm* labour (the stage that directly precedes the delivery). However, in spite of these studies, there is no internationally agreed definition of *preterm* labour. Nonetheless, in practice, women who experience regular contractions, increased vaginal discharge, pelvic pressure and lower backache tend to show threatening preterm labour (*TPL*). While this is a good measure, Mangham *et al.*, suggest that clinical methods for diagnosing *preterm* labour are insufficient [4]. Following a medical diagnosis of *TPL*, only 50% of all women with *TPL* actually deliver, within seven days [2]. In support of this, McPheeters *et al.*, carried out a similar study that showed 144 out of 234 (61.5%) women diagnosed with *preterm* labour went on to deliver at *term* [7]. This can potentially add significant costs, and unnecessary interventions, to prenatal care. In contrast, false-negative results mean that patients requiring admittance are turned away, but actually go on to deliver prematurely [8].

Predicting *preterm* birth and diagnosing *preterm* labour clearly have important consequences, for both health and the economy. However, most efforts have concentrated on mitigating the effects of *preterm* birth. Nevertheless, since this approach remains costly [1], it has been suggested that prevention could yield better results [9]. Effective prediction of *preterm* births could contribute to improving prevention, through appropriate medical and lifestyle interventions. One promising method is the use of Electrohysterography (*EHG*). *EHG* measures electrical activity in the uterus, and is a specific form of electromyography (*EMG*), the measurement of such activity in muscular tissue. Several studies have shown that the *EHG* record may vary from woman to woman, depending on whether she is in true labour or false labour and whether she will deliver *term* or *preterm. EHG* provides a strong basis for objective predication and diagnosis of *preterm* birth.

Many research studies have used *EHG* for prediction or detection of true labour. In contrast, this paper focuses on using *EHG* classification to determine whether delivery will be *preterm* or *term*. This is achieved by comparing various machine-learning classifiers against an open dataset, containing 300 records (38 *preterm* and 262 *term*) [10], using a signal filter and pre-selected features, which are suited to classifying *term* and *preterm* records. The results indicate that the selected classifiers outperform a number of approaches, used in many other studies.

The structure, of the remainder, of this paper is as follows. Section 2 describes the underlying principles of Electrohysterography. Section 3 describes how features are extracted from Electrohysterography signals. Section 4 discusses machine learning and its use in *term* and *preterm* classification, while section 5 presents the approach taken in this paper. Section 6 describes the evaluation, and Section 7 discusses the results. Section 8 then concludes the paper.

## Analysis and Methods

### Electrohysterography

Electrohysterography (EHG) is the term given for the recording of electrical activity of the uterus, in the time domain. In order to retrieve *EHG* signals, bipolar electrodes are adhered to the abdominal surface. These are spaced at a horizontal, or vertical, distance of 2.5 cm to 7 cm apart. Most studies, including [10], use four electrodes, although one study utilizes two [11]. In a series of other studies, sixteen electrodes were used [12]–[17], and a high-density grid of 64 small electrodes were used in [18]. The results show that *EHG* may vary from woman to woman. This is dependent on whether she is in true or false labour, and whether she will deliver at *term*, or prematurely.

A raw *EHG* signal results from the propagation of electrical activity, between cells in the myometrium (the muscular wall of the uterus). This signal measures the potential difference between the electrodes, in a time domain. The electrical signals are not propagated by nerve endings; however, the propagation mechanism is not clear [19]. Since the late 70s, one theory suggests that gap junctions are the mechanisms responsible. Nevertheless, more recently it has been suggested that interstitial cells, or stretch receptors may be the cause of propagation [20]. Gap junctions are groups of proteins that provide channels of low electrical resistance between cells. In most pregnancies, the connections between gap junctions are sparse, although gradually increasing, until the last few days before labour. A specific pacemaker site has not been conclusively identified, although, due to obvious physiological reasons, there may be a generalised propagation direction, from the top to the bottom of the uterus [21].

The electrical signals, in the uterus, are ‘commands’ to contract. During labour, the position of the bursts, in an *EHG* signal, corresponds roughly with the bursts shown in a tocodynamometer or intrauterine pressure catheter (*IUPC*). Clinical practises use these devices to measure contractions. More surprisingly, distinct contraction-related, electrical uterine activity is present early on in pregnancy, even when a woman is not in true labour. Gondry *et al*. identified spontaneous contractions from *EHG* records as early as 19 weeks of gestation [22]. The level of activity is said to increase, as the time to deliver nears, but shoots up especially so, in the last three to four days, before delivery [23]. As the gestational period increases, the gradual increase in electrical activity is a manifestation of the body's preparation for the final act of labour and parturition. In preparation for full contractions, which are needed to create the force and synchronicity required for a sustained period of true labour, the body gradually increases the number of electrical connections (gap junctions), between cells. In turn, this produces contractions in training.

Before analysis or classification occurs, *EHG* signals, in their raw form, need pre-processing. Pre-processing can include filtering, de-noising, wavelet shrinkage or transformation and automatic detection of bursts. Recently, studies have typically focused on filtering the *EHG* signals to allow a bandpass between 0.05 Hz and 16 Hz [24]–[28]. However, there are some that have filtered *EHG* recordings as high as 50 Hz [19]. Nevertheless, using *EHG* with such a wide range of frequencies is not the recommended method, since more interference affects the signal.

### Feature Extraction from Elecrohysterography

The collection of raw *EHG* signals is always temporal. However, for analysis and feature extraction purposes, translation, into other domains, is possible and often required. These include frequency representation, via Fourier Transform, [15], [28]–[30] and wavelet transform [24], [27], [30]–[33]. The advantage of frequency-related parameters is that they are less susceptible to signal quality variations, due to electrode placement or the physical characteristics of the subjects [26]. In order to calculate these parameters, a transform from the time domain is required, *i.e.*, using a Fourier transform of the signal. In several of the studies reviewed, in order to obtain frequency parameters, Power Spectral Density (*PSD*) is used. *Peak frequency* is one of the features provided within the Term-Preterm ElectroHysteroGram (TPEHG) dataset, used within this paper. It describes the frequency of the highest peak in the *PSD*. Most studies focus on the *peak frequency* of the burst, in both human and animal studies, and is said to be one of the most useful parameters for predicting true labour [34]. On the other hand, the study by [10] found *medium frequency* to be more helpful in determining whether delivery was going to be *term* or *preterm*.

Several studies have shown that *peak frequency* increases, as the time to delivery decreases; generally, this occurs within 1–7 days of delivery [11], [19], [24], [26], [30], [35]. In particular, the results in [28] show that there are, statistically, significant differences in the *mean* values of *peak frequency* and the *standard deviations* in *EHG* recordings taken during *term* labour (*TL*) and *term* non-labour (*TN*) and also between *preterm* labour (*PTL*) and *preterm* non-labour (*PTN*).

In comparison to *peak frequency*, the TPEHG study [10] found that *median frequency* displayed a more significant difference, between *term* and *preterm* records. When considering all 300 records, the statistical significance was p = 0.012 and p = 0.013, for *Channel 3*, on the 0.3–3 Hz and 0.3–4 Hz filter, respectively. Furthermore, this significance (p = 0.03) was also apparent when only considering early records (before 26 weeks of gestation), with the same 0.3–3 Hz filter, on *Channel 3*. The TPEHG study [10] concluded that this might have been due to the enlargement of the uterus, during pregnancy, which would affect the position of electrodes. The placement of the *Channel 3* electrode was, approximately, always 3.5cm below the navel. However, as pregnancy progressed, this would mean that the electrode would move further away from the bottom of the uterus (cervico-isthmic section). If a generalised pacemaker area actually exists, and it is at the cervico-isthmic section, then, as pregnancy progresses, its position would move further and further away from the electrode, resulting in a diminished record of the signal. Whether this explanation is true or not, the results of [10] show that, the discriminating capability of *median frequency* is somehow diminished, after the 26th week.

Amplitude-related *EMG* parameters represent the uterine *EMG* signal power, or signal energy. However, a major limitation is that the differences in patients can easily affect these parameters. Patients may differ in the amount of fatty tissue they have, and the conductivity of the skin–electrode interface, which leads to differences in the attenuation of uterine signals [8], [26], [34]. Examples of amplitude-related parameters include *root mean square, peak amplitude* and *median amplitude*.

Using the *Student's t-test*, [10] found that *root mean square* might be useful in distinguishing between whether the information was recorded early (before 26 weeks of gestation) or late (after 26 weeks). The results obtained are in agreement with [19], [30] and [36], who found that the amplitude of the power spectrum increased, just prior to delivery. This was despite only analysing the *root mean square* values, per burst, rather than the whole signal. On the other hand, other studies did not find that amplitude-related parameters displayed a significant relationship to gestational age or indicate a transition to delivery (within seven days) [23], [25], [28]. Some of these discrepancies may be due to the differences between the characteristics in the studies: [10] compared records before and after 26 weeks, whereas [25] only examined records after the 25th week; [29] and [35] studied rat pregnancy, in contrast to human pregnancy. The frequency band used in [30] and [19] was also a much broader band than in other studies (0.3–50 Hz; no bandwidth given for [36]), and the studies by [29] and [35] measured per burst, whilst [25] measured the whole signal.

Meanwhile, the TPEHG study [10] could not find any significant difference in *root mean squares* between *preterm* and *term* records. However, [25] did find that the *root mean squares*, in *preterm* contractions, were higher (17.5 mv ±7.78), compared to *term* contractions (12.2 mV ±6.25; p<0.05). The results, from [25], could not find a correlation between *root mean squares* and the weeks left to delivery. Nevertheless, they do suggest that a greater *root mean square* value was, for the most part, a static symptom that indicated a woman's dispensation to give birth prematurely. They also found that the *root mean square* values, within each pregnancy, did increase within a few days of birth.

Overall, the results suggest that there is no significant difference in the amplitude-related parameters between *term* and *preterm* deliveries, when taken during labour, or close to it. However, there may be considerable differences earlier on in the pregnancy. This suggests that by the time of delivery, any differences have equalised themselves.

*Sample entropy* measures the irregularity of a time series, of finite lengths. This method was introduced by [37] to measure complexity in cardiovascular and biological signals. The more unpredictable the time series is, within a signal recording, the higher its sample entropy. The process is based on calculating the number of matches of a sequence, which lasts for *m* points, within a given margin *r*. The disadvantage of this technique is the requirement to select two parameters, *m* and *r*. However, *sample entropy* did show a statistical difference between *term* and *preterm* delivery information, recorded either before or after the 26th week of gestation, when using any of the filters, but only using the signal from *Channel 3* [10].

### Term and Preterm Classification

Computer algorithms, and visualization techniques, are fundamental in supporting the analysis of datasets. More recently, the medical domain has been using such techniques, extensively.

Artificial Neural Networks (*ANN*) have been used in a large number of studies to classify *term* and *preterm* deliveries, [11], [38]. They have also been useful for distinguishing between *non-labour* and *labour* events [11], [38], irrespective of whether they were *term* or *preterm*. Moslem *et al.* [14] argue that they have been particularly useful in helping to identify important risk factors associated with *preterm* birth. The global accuracy of these studies varied from between 73% and 97%.

Baghamoradi *et al.* [39] used the TPEHG database [10] to compare sample entropy with thirty and three cepstral coefficients extracted from each signal recording through sequential forward selection and Fisher's discriminant. A multi-layer perceptron (MLP) neural network classified the feature vectors into *term* and *preterm* records. The results indicate that the three cepstral coefficients produced the best classification accuracy, with 72.73% (±13.5), while using all thirty coefficients showed only 53.11% (±10.5) accuracy. S*ample entropy* performed the worst with an accuracy of 51.67% (±14.6). The results indicate that the sequential forward selection and Fisher's discriminant had the most effect on the accuracy because the thirty coefficients set only presenting a small improvement, in classification accuracy.

Support Vector Machines (*SVM*) have featured in several studies, which include [12], [13], [14]. Many of them classify contractions into labour or non-labour, using different locations on the abdomen. Majority voting (*WMV*) decision fusion rules, including a Gaussian radial basis function (*RBF*), form the basis for classification. The feature vectors include the *power* of the *EMG* signal, and the *median frequency*. The highest accuracy for a single *SVM* classifier, at one particular location on the abdomen, was 78.4% [12], [13], whilst the overall classification accuracy, for the combined *SVM*, was 88.4% [14]. Finding the coefficients, for the decision boundary, occurs by solving a quadratic optimisation problem.

The *k-NN* algorithm has been used by Diab *et al.* [40] with an emphasis on Autoregressive (*AR*) modelling and wavelet transform pre-processing techniques. The study focused on classifying contractions into three types using data obtained from 16 women. Group 1 (G1), were women who had their contractions recorded at 29 weeks, and then delivered at 33 weeks; Group 2 (G2) were also recorded at 29 weeks, but delivered at 31 weeks, and Group 3 (G3) were recorded at 27 weeks and delivered at 31 weeks. Classification occurred against G1 and G2 and against G2 and G3 using, the *k-NN* algorithm combined with the pre-processing method of *AR*. As well as this, an Unsupervised Statistical Classification Method (*USCM*), combined with the pre-processing method of Wavelet Transform, was also used. The *USCM* adopted the *Fisher Test and k-Means* methods. The wavelet transform, combined with *USCM*, provided a classification error of 9.5%, when discerning G1 against G2, and 13.8% when classifying G2 against G3. Using *AR*, the *k-NN* provided a classification error of 2.4% for G1 against G2 and 8.3% for G2 against G3. In both classifications, the *AR* and *k-NN* methods performed better than the *USCM*. Furthermore, the classification accuracy for G1 and G2 was always lower than the equivalent G2 and G3 classifications. This suggests that it is easier to distinguish between pregnancies recorded at different stages of gestation than it is to distinguish between the time of delivery.

### Methodology

Despite the advances, within the last twenty years, in the *EHG* diagnosis and prediction field, knowledge of the uterus, and its mechanisms, remains relatively poor. This is especially evident when compared to other organs, such as the heart, and to a lesser extent, the gastro-intestinal system [20]. Given this inadequate knowledge, it may be easier to utilise an empirical backward looking, ‘data mining’ or ‘brute force’ approach. This is opposed to a forward-looking, conceptual model approach, in order to find features that best describe pregnancy.

The aim of most studies, in *EHG* prediction or detection, has been to detect true labour, rather than predicting, in advance, whether delivery will be *preterm* or *term*. Furthermore, many of the studies concentrated on a late state in gestation. Even when earlier stages are incorporated, they always only included those with threatened *preterm* labour. However, the TPEHG dataset is different, as it involves the general population of pregnant women. Therefore, this collection includes fewer records for women who delivered *preterm* than *term*.

For *term* deliveries, true labour only starts within 24 hours. For *preterm* deliveries, it may start within 7 to 10 days. The change in *EHG* activity, from non-labour to labour, is dramatic; throughout the rest of the pregnancy, any change in *EHG* is more gradual. Therefore, classification of records, into *preterm* and *term*, is particularly challenging. For this reason, and due to the configuration of the dataset, the study attempts to classify records from an earlier stage, according to whether they will eventually result in *term* or *preterm* deliveries.

Fele-Zorz *et al*. conducted a comprehensive study that compared linear and non-linear signal processing techniques to separate uterine *EMG* records of *term* and *preterm* delivery groups [10]. The *EHG* records are from a general population of pregnant patients at the Department of Obstetrics and Gynaecology Medical Centre in Ljubljana, gathered between 1997 and 2006. These records are publicly available, via the TPEHG dataset, in Physionet.

The TPEHG dataset contains 300 records (one record per pregnancy). Each recording is approximately 30 minutes long. Records are either recorded early, <26 weeks (at around 23 weeks of gestation) or later,  = >26 weeks (at around 31 weeks). It is not clear why the 26^th^ week is used as the dividing line for early and late records, however, this is possibly because of significant changes that occur in the 3^rd^ trimester of pregnancy. Table 1, below, shows the classification of records in the TPEHG dataset.

**Table 1:** Numbers of Patients in each group.

Terms:	*Term Deliveries*	*Term Deliveries*	*Preterm Deliveries*	*Preterm Deliveries*	*All Deliveries*	*All Deliveries*
Recording Time	Number of records	Mean/Median Recording weeks	Number of records	Median/Median Recording Weeks	Number of records	Mean/Median Recording Weeks
**Early**	143	22.7/22.86	19	23.0/23.43	162	22.73/23.0
**Later**	119	30.8/31.14	19	30.2/30.86	138	30.71/31.14
**All Recording Time**	262	26.75/24.36	38	27.0/25.86	300	26.78/24.43

The recording time relates to the gestational age of the foetus, at the time of the recoding. The classifications of these recordings, as *term* and *preterm* deliveries, was made retrospectively, after giving birth, and following the widely used definition of preterm being under a fully completed 37 weeks. Therefore, the four categories of recordings are as follows:

1. Early-Term: Recordings made early, which resulted in a term delivery.
2. Early-Preterm: Recordings made early, which resulted in a preterm delivery.
3. Late-Term: Recordings made late, which resulted in a term delivery.
4. Late-Preterm: Recordings made late, which resulted in a preterm delivery.

**Figure 1:**
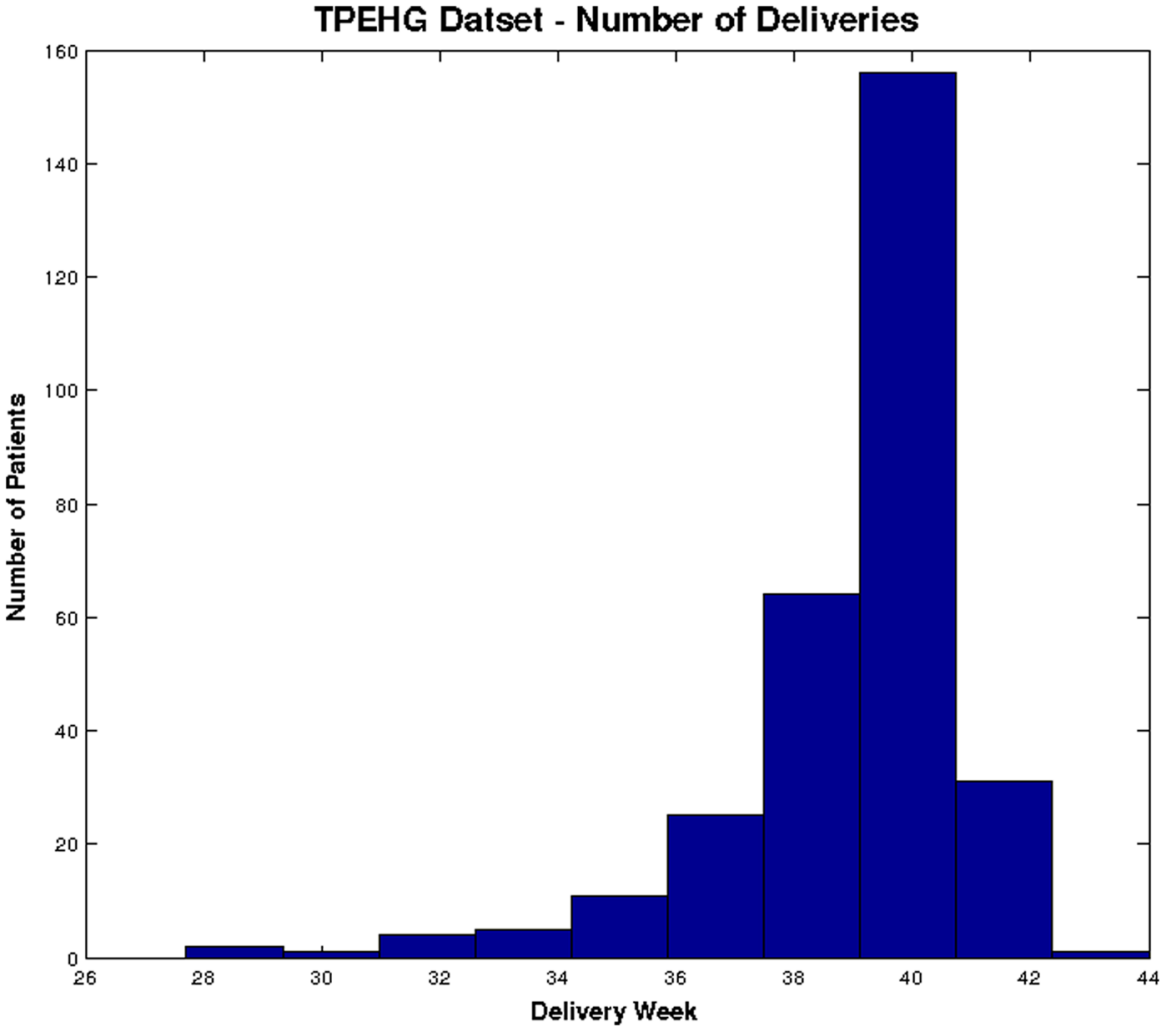
Distribution of deliveries in TPEHG dataset.

Figure 1 shows the distributions of *term* and *preterm* records in the TPEHG dataset, which clearly indicates that the majority of the data are *term*.

In summary, this paper uses 300 records that consist of ‘*38*’ *preterm* and ‘262’ *term* records.

### Data Pre-processing

In the TPEHG dataset, the records have a sample frequency of 20 Hz, and 16-bit resolution, with an amplitude range of ±2.5 mV. Before sampling took place, an analogue, three-pole, Butterworth filter, filtered the signal within the range of 1–5 Hz. Signals were recorded simultaneously through three different channels (Channel1, Channel2, and Channel3), via four electrodes attached to the abdominal surface, with the navel at the symmetrical centre.

Fele-Zorz *et al.* showed that the 0.3–3 Hz filtered signals on *Channel 3* is the best filter for discriminating between *preterm* and *term* delivery records [10]. The results show that *sensitivities* (true positives – in this instance preterm records), produced by several of the classifiers, was higher than those produced when other filters were used [10]. However, there was no appropriate filter to remove unwanted artefacts, such as maternal heart rate. Uterine activity has been found to comprise both ‘fast’ and ‘slow’ signals of high and low frequency signals. The fast waves represent the individual electrical signals firing, whilst the slow waves correspond to the resulting mechanical contractions. Slow waves exist between 0.03 and 0.3 Hz, and the fast waves exist between 0.3 and 3.0 Hz. Reference [36] found in a study of 99 pregnant patients, that 98% of uterine electrical activity occurred in frequencies less than 1 Hz, and that the maternal heart rate (*ECG*) was always higher than 1 Hz. Furthermore, 95% of the patients, measured had respiration rates of 0.33 Hz or less. Therefore, the authors considered that a 0.34–1 Hz bandpass filter removed most of the unwanted artefacts. Several other studies have adopted the same filtering scheme [53]–[54], and [12]. Therefore, in this paper, the raw Channel 3 signal was chosen and filtered using a 0.34–1 Hz filter. This is to coincide with the findings in [10] and [36].

### Features Selection

**Figure 2:**
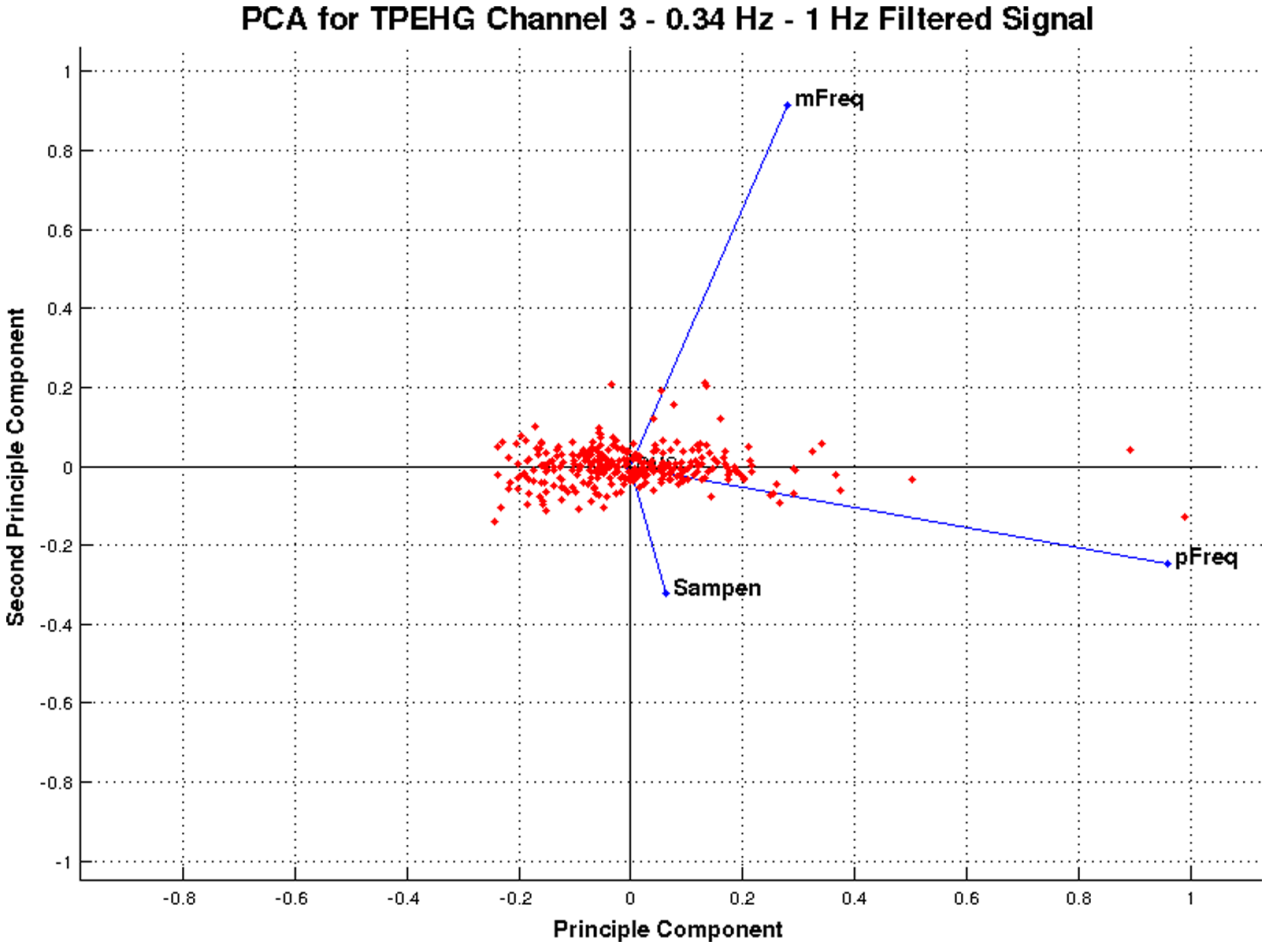
PCA for features extracted from the Channel 3 0.34–1 Hz filtered signal.

The feature vectors in this paper are generated using four features – *root mean squares, peak frequency, median frequency,* and *sample entropy*. The literature reports that *Mean frequency* and *sample entropy* have the most potential to discriminate between *term* and *preterm* records. However, *root mean squares* and *peak frequencies* have had conflicting results. Nonetheless, several studies report that these features are useful for discriminating between *term* and *preterm* records. To validate these findings, the discriminant capabilities of each feature are determined using principal component analysis (*PCA*). Figure 2 shows the *PCA* for the features extracted from *Channel 3* 0.34–1 Hz filter signal.

As indicated in Figure 2, the horizontal axis shows that the peak frequency is the principal component and has the most discriminant capabilities of the four features considered. This is consistent with the findings in [11], [19], [24], [26], [28], [30], [35]. The vertical axis shows median frequency as the second component with very good discriminant capabilities. This is consistent with the findings in [10]. *Sample entropy* is the third component and hence considered useful. These findings are broadly consistent with [10], which found a statistical difference between *term* and *preterm* records, using *sample entropy*. Finally, the root mean squares feature resides towards the cross-section of the first and second components, as indicated in Figure 2. This feature has the least discriminative capabilities and again the findings are consistent with [10]. Nevertheless, [25] suggested that the *root mean square* is a useful feature because, in *preterm* contractions, it is higher.

In summary *PCA*, in conjunction with various studies reported in the literature, make a very strong case for the use of *peak frequency, median frequency, root mean squares* and *sample entropy* in discriminating between *term* and *preterm* records.

### Synthetic minority over-sampling

In a two class balanced dataset the prior probabilities will be equal for each. This is not the case for the TPEHG dataset because it is not balanced. There are 262 true negatives (majority class) and 38 true positive values (minority class). Classifiers are more sensitive to detecting the majority class and less sensitive to the minority class and this leads to biased classification [1]. Therefore, given a random sample taken from the dataset, the probability of a classifier classifying a pregnant woman as *term* will be much higher (87.3%–262/300) than the probability of it classifying a pregnant woman as *preterm* (12.6%–38/300). This imposes a higher cost for misclassifying the minority (predicting that a pregnant woman is likely to deliver full term only to go home and deliver prematurely) than the majority class, (predicting a pregnant woman will deliver preterm only to go deliver at term).

In order to address this problem, it is necessary to resample the dataset. Various resampling techniques are available, and these include under sampling and over sampling [3]. Under sampling reduces the number of records from the majority class to make it equal to the minor class – in this instance it would mean removing 224 records leaving us with a small dataset. Data in the minority class is generated using oversampling. In this study, the synthetic minority over-sampling technique (SMOTE) is used rather than reducing the dataset further [41].

**Figure 3:**
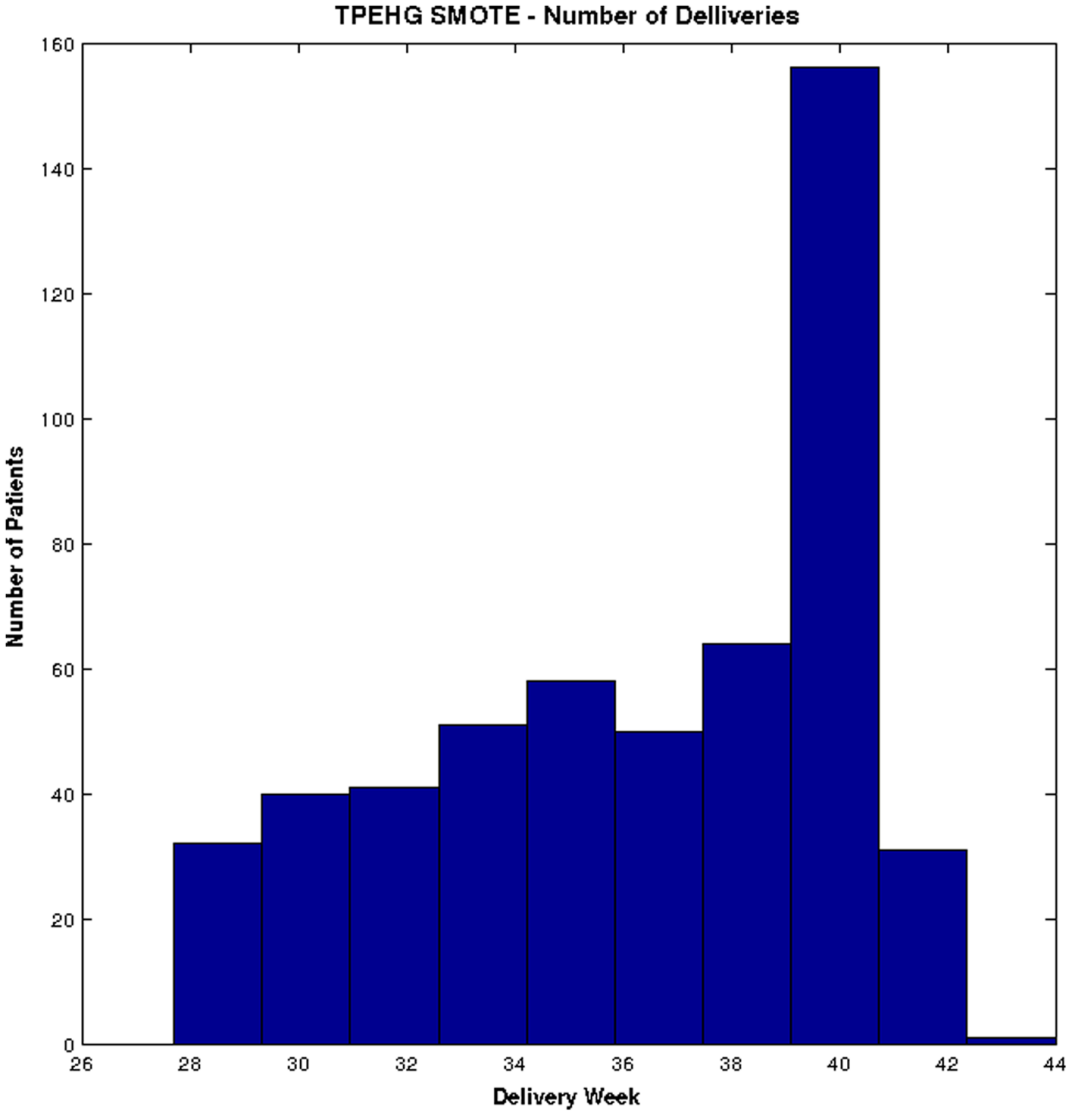
Distribution of deliveries in TPEHG dataset after the SMOTE technique is applied.

Several studies have shown that the SMOTE technique effectively solves the class skew problem [42]–[47]. Using SMOTE, the minority class (*preterm*) is oversampled using each minority class records, in order to generate new synthetic records along line segments joining the *k* minority class nearest neighbours. This forces the decision region of the minority class to become more general and ensures that the classifier creates larger and less specific decision regions, rather than smaller specific regions. In [41] the authors indicated that this approach is an accepted technique for solving the problems related to unbalanced datasets. Figure 3 shows the distribution of *term* and *preterm* records, using the SMOTE technique.

Figure 3 clearly shows that using the SMOTE technique allows the *term* and *preterm* dataset to be more balanced, compared to the original TPEHG dataset.

### Classification

Following an analysis of the literature, the study in this paper uses simple, yet powerful algorithms, as shown in Table 2.

**Table 2:** Summary of Classifiers, Features, Validation Techniques and Sample Sizes used in this study.

Classifiers	Features	Validation	Sample Sizes
***Density-Based***	Root Mean Squares	Holdout Cross Validation	Original (38 preterm/262 term)
Linear Discriminant Classifier (LDC)	Peak Frequency	k-fold Cross Validation	SMOTE (262 preterm/262 term)
Quadratic Discriminant Classifier (QDC)	Median Frequency	Sensitivity/Specificity	SMOTE Clinical (150 preterm/150 term)
Uncorrelated Normal Density Classifier (UDC)	Sample Entropy	Receiver Operator Curve	Clinical (38 preterm/262 term)
***Linear and Polynomial-Based***		Area Under the Curve	
Polynomial Classifier (POLYC)			
Logistic Classifier (LOGLC)			
***Nonlinear-Based***			
*K* Nearest Neighbour Classifier (KNNC)			
Decision Tree Classifier (TREEC)			
Parzen Classifier (PARZENC)			
Support Vector Classifier (SVC)			

The classifiers considered in this study include the linear discriminant classifier (*LDC*), quadratic discriminant classifier (*QDC*), uncorrelated normal density based classifier (*UDC*), polynomial classifier (*POLYC*), logistic classifier (*LOGLC*), 3-NN (*KNNC*), decision tree (*TREEC*), parzen classifier (*PARZENC*) and the support vector classifier (*SVC*) [48]. The linear, quadratic and uncorrelated normal density-based classifiers are all density-based classifiers. The *LDC* is particularly useful when two classes are not normally distributed, and where monotonic transformations, of posterior probabilities, helps to generate discriminant functions. The *QDC* assumes that the classes are normally distributed with class specific covariance matrices, thus allowing a set of optimal discriminant functions to be obtained. The *UDC* works in a similar way to the *QDC* classifier but computation of a quadratic classifier between the classes by assume normal densities with uncorrelated features. The *QDC* takes decisions by assuming different normal distribution of data that leads to quadratic decision boundaries.

The polynomial and logistic classifiers are linear-based classifiers, which predict class labels based on weighted, linear combination of features or the variables of the objects. The *LOGLC* computes the classification of a dataset by maximizing the likelihood criterion, using the logistic (sigmoid) function. The *POLYC* adds polynomial features to the datasets in order to run the untrained classifier. It is possible to construct second order terms, using this classifier. The parzen, decision tree, support vector, and *k-*nearest neighbour classifiers are nonlinear classifiers. Nonlinear classifiers compute the optimum smoothing parameter between classes in the datasets. Using smoothing parameters without any learning process, produces discrimination. Smoothing parameters may be a scalar, a vector or a matrix with objects and their features. The *TREEC* classifier uses binary splitting and classes are decided upon the basis of a sequence of decision rules. Quadratic programming optimises the *SVC*, and non-linearity is determined by the kernel. If an *SVM* model, uses the sigmoid kernel then it behaves more or less like a two-layer, perceptron neural network. There are four basic kernels, linear, polynomial, radial basis function and sigmoid. In this type of classification, functions map training sets into a higher dimensional space in this type of classifier. It finds a linear separating hyperplane with the maximum margin in the higher dimensional space. The *KNNC* and *PARZENC* are similar in the sense that their build-up classifiers still use the training dataset and their parameters, while *KNNC* classifies the object in a feature space with the nearest training parameters.

### Validation Methods

The **Holdout Cross-Validation** technique is used in this study [49], in which, 80% of the whole dataset is designated for training and the remaining 20% for testing. To maintain generalisation, the training and test sets comprise randomly selected instances from the TPEHG dataset. Since the exact selection of instances, for the training, is random, it is necessary to repeat the learning and testing stage. The average performance obtained from 100 simulations is utilised. This number is considered, by statisticians, to be an adequate number of iterations to obtain an average [50]. After each repetition, the error rate for each classifier is stored and the learning experience of the algorithm wiped so that it does not influence the next test. Producing several repetitions provides average error rates, standard deviations and performance values for each classifier.

The ***k-fold*** **cross-validation** is a validation technique used to estimate the accuracy of the classifiers. In this paper, the results obtained for *k-fold* validation uses 5 folds and 1 and 100 repetitions respectively. The results are then compared with those from the 80/20 holdout cross-validation approach. ***Sensitivity*** (true positives) and ***specificity*** (true negatives) measure the predictive capabilities of classifiers in binary classification tests. *Sensitivities* refer to the true positive rate or recall rate (preterm records). *Specificities* measure the proportion of true negatives (term records). *Sensitivities* are considered a higher priority than *specificities,* in this study. It is important to predict a preterm delivery rather than miss classifying a term pregnancy.

The ***Receiver Operator Curve*** **(ROC)** is a standard technique used to summarise classifier performance based on trade-offs between true positive and true negative error rates [51]. The ***Area Under the Curve*** **(AUC)** is an accepted performance metric that provides a value equal to the probability that a classifier will rank a randomly chosen positive instance higher than a randomly chosen negative one (this obviously assumes that positive ranges higher than negative) [51]. These have been chosen since they are suitable evaluation methods for classifiers, which produce binary output (*term* or *preterm*) [52].

The pattern recognition toolbox (PRTools) has been used to implement all of the techniques used in this study.

## Results

This section presents the classification results for *term* and *preterm* delivery records using the TPEHG dataset. The 0.34–1 Hz filter on *Channel 3* is used with 80% *holdout* technique and *k-fold* cross-validation. The initial evaluation provides a base line for comparison against all subsequent evaluations, considered in this section.

### Results for 0.34–1 Hz TPEHG Filter on Channel 3

This evaluation uses the 0.34–1 Hz filtered signals on Channel 3 with nine classifiers. The performance for each classifier is evaluated, using the *sensitivity, specificity, mean error, standard deviation* and *AUC* values with 100 simulations and randomly selected training and testing sets for each simulation.

### Classifier Performance

The first evaluation uses the original TPEHG dataset (38 *preterm* and 262 *term*). Table 3, illustrates the mean averages obtained over 100 simulations for the *sensitivity, specificity,* and *AUC*.

**Table 3:** Classifier Performance Results for the 0.34–1 Hz Filter.

	Sensitivity	Specificity	AUC
Classifier	*Channel 3 0.34–1Hz Filter*	*Channel 3 0.34–1Hz Filter*	*Channel 3 0.34–1Hz Filter*
*LDC*	0.0000	0.9807	53%
*QDC*	0.0000	0.9807	53%
*UDC*	0.0000	1.0000	52%
*POLYC*	0.0000	0.9807	61%
*LOGLC*	0.0000	0.9807	60%
*KNNC*	0.0000	0.9230	53%
*TREEC*	0.2857	0.8653	60%
*PARZENC*	0.0000	1.0000	50%
*SVC*	0.0000	1.0000	61%

As shown in Table 3, the *sensitivities* (*preterm*), in this initial test, are low for all classifiers. This is expected because there are a limited number of *preterm* records from which the classifiers can learn. Consequently, *specificities* are higher than *sensitivities*. More specifically, there are 31 *preterm* records in the 80% *holdout* training set. This is a limited number of records for one class. Furthermore, the *AUC* indicated that all classifiers failed to generate results higher than 61%. This indicates that the classifiers classified most of the instances into the major class, which caused very low sensitivities. Table 4 illustrates the results from a *k-fold* cross-validation technique, used to improve the results obtained from the holdout method. The results showed that it was not possible to achieve a classification error, lower than the base-rate error of 12.67%.

**Table 4:** Cross Validation Results for the 0.34–1 Hz Filter.

	80% Holdout: 100 Repetitions		Cross Val, 5 Folds, 1 Repetitions	Cross Val, 5 Folds, 100 Repetitions	
Classifiers	*Mean Err*	*SD*	*Mean Err*	*Mean Err*	*SD*
*LDC*	0.1342	0.0127	0.1333	0.1349	0.0045
*QDC*	0.1355	0.0166	0.1366	0.1421	0.0088
*UDC*	0.1324	0.0142	0.1366	0.1383	0.0080
*POLYC*	0.1300	0.0072	0.1300	0.1300	0.0000
*LOGLC*	0.1324	0.0112	0.1333	0.1322	0.0034
*KNNC*	0.1707	0.0270	0.1267	0.1312	0.0081
*TREEC*	0.2135	0.0443	0.1995	0.2183	0.0210
*PARZENC*	0.1267	0.0000	0.1267	0.1267	0.0000
*SVC*	0.1267	0.0000	0.1267	0.1267	0.0000

The *k-fold* cross-validation results, using five folds and both *one* and *one hundred* repetitions shows that the *k-fold* cross-validation approach improved the error rates, for some classifiers. However, these results are not considered statistically significant. Furthermore, the lowest error rates could not be improved below the minimum error rate expected, which is 12.67% (38 preterm/300 deliveries).

### Model Selection

**Figure 4:**
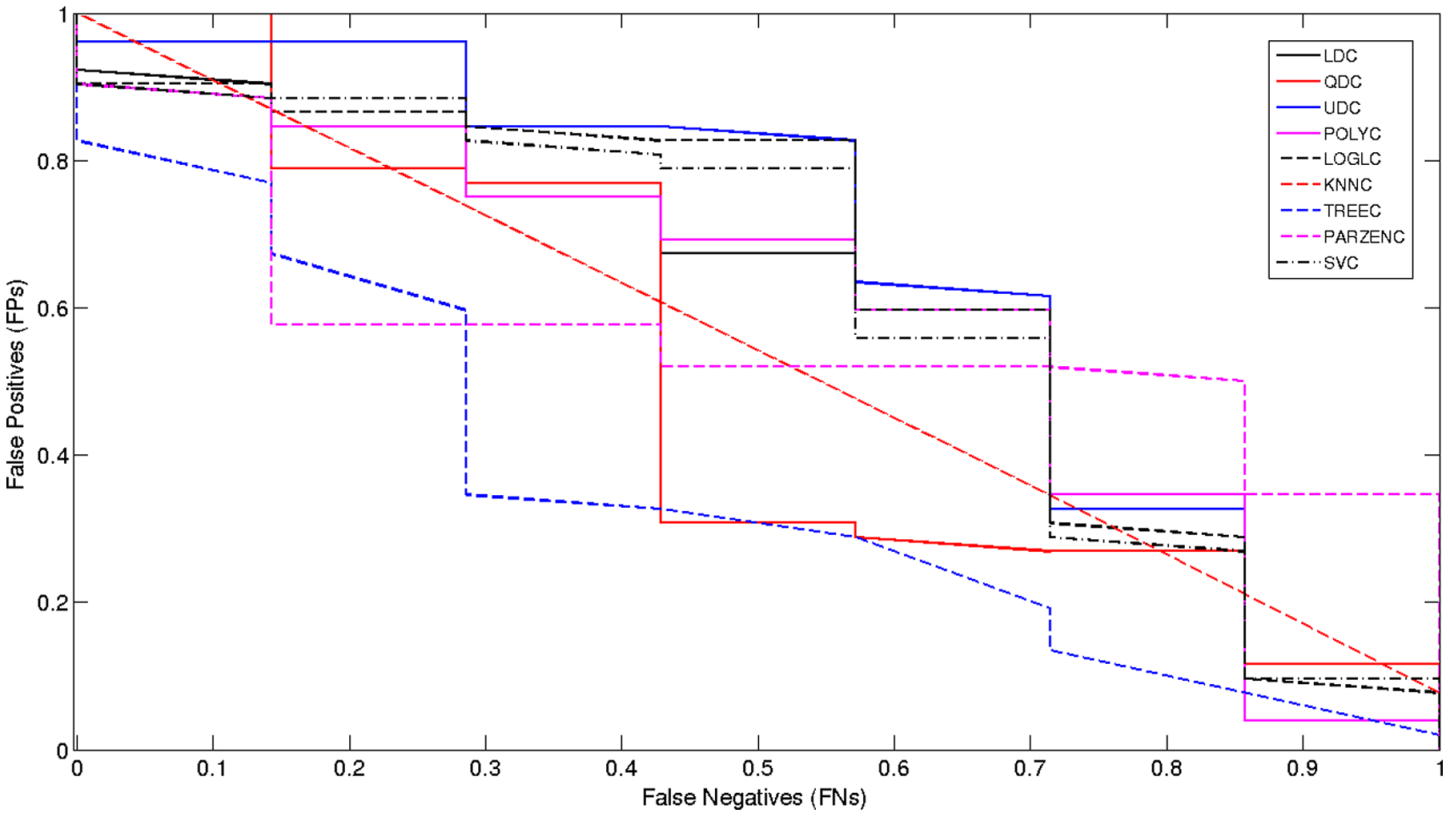
Received Operator Curve for the 0.34–1 Hz Filter.

The receiver operator characteristic (*ROC*) curve shows the cut-off values for the *false negative* and *false positive* rates. It has been used for each of the classifiers, using the original TPEHG dataset 0.34–1 Hz filter. Figure 4 indicates that, none of the classifiers performed particularly well. The *AUC* values in Table 1 support these findings with very low accuracy values.

The poor results indicate that the classification algorithms do not have enough *preterm* records to learn from, in comparison to *term* records. Consequently, *sensitivities* are low while *specificities* are high, which in this study are of lower importance. The main issue, in terms of machine learning, is that the dataset is skewed. Although this problem has not been widely reported, in many recent *EHG* studies, imbalanced data is a common machine-learning problem. As such, re-sampling the classes (with the minority class – in this instance, preterm records) is a conventional way to balance the dataset [53].

### Results for 0.34–1 Hz TPEHG Filter on Channel 3 – Oversampled using SMOTE

The 38 *preterm* records are re-sampled using the SMOTE technique [41]. The SMOTE algorithm allows a new dataset to be generated that contains an even split between *term* and *preterm* records (262 each) oversampled using the original preterm records.

### Classifier Performance

Table 5 indicates that the *sensitivities*, for all the algorithms, improved at the expense of lower *specificities*. In addition, the *AUC* results showed significant improvements with a value of 89% achieved by the *TREEC* classifier. The results also show that the *AUC* values, for all the algorithms, increased. This is encouraging given that *sensitivities* are more important in this research than *specificities*. Balancing the dataset increased the classification algorithms ability to predict *preterm* records. From the previous set of results, we find a 60% increase in *sensitivities*, a 17% drop in *specificities*, and a 30% increase in the performance of the *TREEC* classifier.

**Table 5:** Classifier Performance Table for Oversampled 0.34–1 Hz Signal.

	Sensitivity	Specificity	AUC
Classifier	*Channel 3 0.34–1Hz Filter*	*Channel 3 0.34–1Hz Filter*	*Channel 3 0.34–1Hz Filter*
*LDC*	0.8653	0.8076	66%
*QDC*	0.9230	0.8461	72%
*UDC*	0.8269	0.8076	72%
*POLYC*	0.8653	0.8076	86%
*LOGLC*	0.8653	0.8269	86%
*KNNC*	0.8653	0.8269	84%
*TREEC*	0.9038	0.8269	89%
*PARZENC*	0.5961	0.9615	72%
*SVC*	0.8076	0.7692	78%

Again, the *k-fold* cross-validation results are better than the holdout method. This is indicated in Table 6.

**Table 6:** Cross Validation Results for Oversampled 0.34–1 Hz Signal.

	80% Holdout: 100 Repetitions		Cross Val, 5 Folds, 1 Repetitions	Cross Val, 5 Folds, 100 Repetitions	
Classifiers	*Mean Err*	*SD*	*Mean Err*	*Mean Err*	*SD*
*LDC*	0.2132	0.0325	0.2116	0.2064	0.0023
*QDC*	0.1770	0.0347	0.1811	0.1806	0.0040
*UDC*	0.2035	0.0328	0.1981	0.2001	0.0018
*POLYC*	0.2132	0.0325	0.2116	0.2064	0.0023
*LOGLC*	0.2037	0.0315	0.2118	0.1972	0.0059
*KNNC*	0.2249	0.0386	0.2594	0.2340	0.0088
*TREEC*	0.1995	0.0387	0.1944	0.1994	0.0069
*PARZENC*	0.2499	0.0392	0.2423	0.2461	0.0124
*SVC*	0.2851	0.0383	0.2899	0.2901	0.0042

The results show that, using the 80% holdout method, several classifiers produce better results. Overall, the mean errors produced, using all of the validation techniques, were significantly lower than the expected error, which is 262/524, i.e. 50%.

### Model Selection

**Figure 5:**
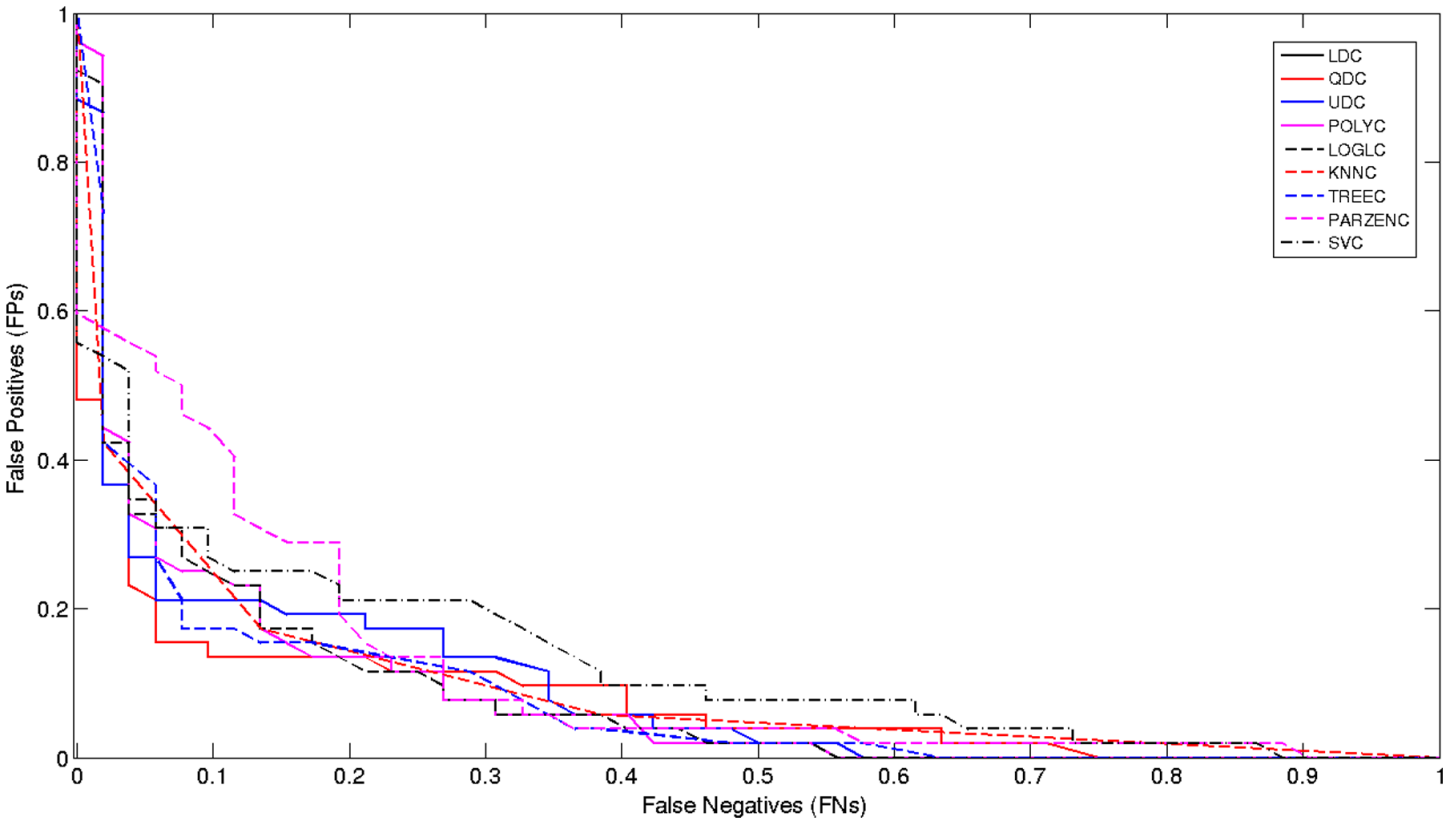
Received Operator Curve for Oversampled 0.34–1 Hz Signal.

Again, the *ROC* curve shows the cut-off values for the *false negative* and *false positive* rates. Figure 5, below, shows a significant improvement.

The results present a strong case for oversampling and indicate that better predictive models are possible for predicting *term* and *preterm* records.

### Results for 0.34–1 Hz TPEHG Filter on Channel 3 – Oversampling with additional features

In December 2012, Fele-Zorz *et al.* made additional features available. These features are *age, parity* (number of previous births), *abortions, weight, hypertension, diabetes, placental position, first* and *second trimester bleeding, funnelling* and *smoking*. Incorporation of the new features, into the original dataset, resulted in the filtration of the dataset. The purpose of this was to remove any noisy data that may have been contained in the additional features. This resulted in a new dataset containing 19 *preterm* records and 150 *term* records. The SMOTE algorithm has balanced the dataset, and the classifiers have been re-run.

### Classifier Performance (Oversampling with additional features)

Table 7 shows the sensitivity, specificity, and AUC results. These results show that there is a significant increase in sensitivity, specificity and AUC values, due to the utilisation of the additional features. The best classification algorithm is the POLYC classifier. This achieved 97% sensitivity, 90% specificity, and 95% AUC value with 8% global error. From the previous set of results, this shows a 6% increase in *sensitivities*, 7% increase in *specificities,* and a 6% increase in the *AUC* value, while maintaining an 8% global error. Other classifiers also produced very good results, particularly, the *LOGLC*, *KNNC* and the *TREEC* classifiers. All these classifiers produced improvements on the classifications performed on the original TPEHG dataset.

**Table 7:** Classifier Performance for Oversampled 0.34–1 Hz Signal with additional Features.

	Sensitivity	Specificity	AUC
Classifier	*Channel 3 0.34–1Hz Filter*	*Channel 3 0.34–1Hz Filter*	*Channel 3 0.34–1Hz Filter*
*LDC*	0.9666	0.9000	70%
*QDC*	0.9666	0.1666	83%
*UDC*	0.9666	0.1333	78%
*POLYC*	0.9666	0.9000	95%
*LOGLC*	0.9666	0.9000	94%
*KNNC*	0.9333	0.8000	90%
*TREEC*	0.9666	0.9000	93%
*PARZENC*	0.9666	0.5666	59%
*SVC*	0.9666	0.7000	92%

The performance of *k-fold* cross-validation was compared with the results obtained from both the 80% *holdout* method and *cross-validation*. Table 8 shows that minor improvements are made, using *k-fold* cross-validation.

**Table 8:** Cross Validation Results for Oversampled 0.34–1 Hz Signal with additional Features.

	80% Holdout: 100 Repetitions		Cross Val, 5 Folds, 1 Repetitions	Cross Val, 5 Folds, 100 Repetitions	
Classifiers	*Mean Err*	*SD*	*Mean Err*	*Mean Err*	*SD*
*LDC*	0.0858	0.0289	0.00800	0.0867	0.0060
*QDC*	0.3260	0.0780	0.0780	0.3344	0.0216
*UDC*	0.4162	0.0471	0.0471	0.4289	0.0124
*POLYC*	0.0858	0.0289	0.0289	0.0867	0.0060
*LOGLC*	0.0932	0.0301	0.0301	0.0983	0.0062
*KNNC*	0.1458	0.411	0.0411	0.1522	0.0131
*TREEC*	0.1127	0.0436	0.0436	0.1178	00.0149
*PARZENC*	0.2130	0.044	0.0444	0.2067	0.0056
*SVC*	0.1338	0.0419	0.0419	0.1233	0.0070

The results show that the additional features significantly improve the performance of several classifiers. In particular, the *POLYC*, *LOGLC, KNNC* and the *TREEC* classifiers perform very well. The best classifier is the *POLYC* with 97% for *sensitivity*, 90% for *specificity*, and an *AUC* value of 95%, with a global mean error of 8%.

### Model Selection

**Figure 6:**
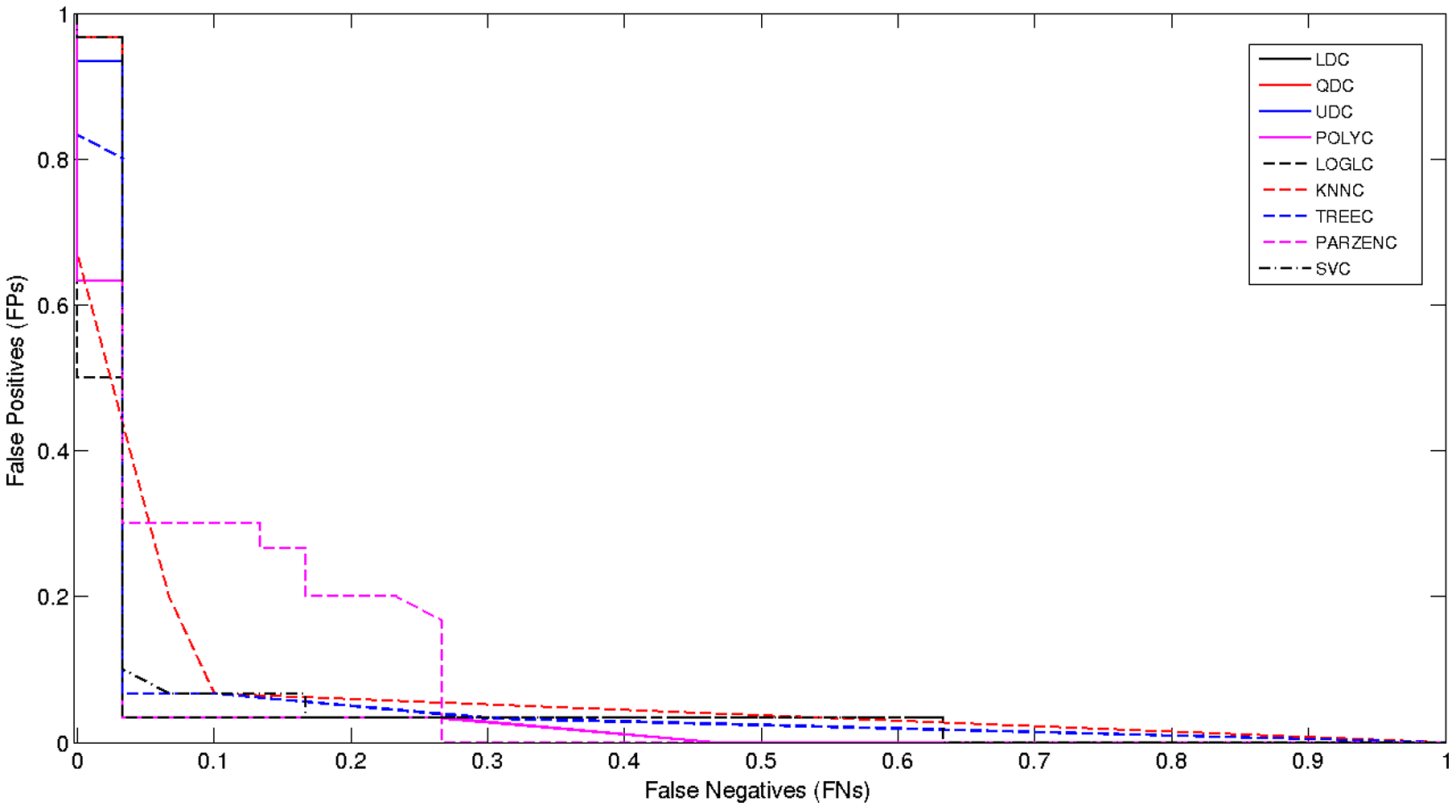
Received Operator Curve for Oversampled 0.34–1 Hz Signal with additional features.

Figure 6 below, shows that there is a significant improvement, compared to the *ROC* curve illustrated in Figure 5.

### Results for Clinical Data Only

In this section, the clinical data by itself is used to classify the *term* and *preterm* records. As before, the dataset is balanced using the SMOTE algorithm. The same classification algorithms have also been re-run, on the new 300 record clinical dataset.

### Classifier Performance (Clinical Data Only)

Table 9 shows the *sensitivity, specificity*, and *AUC* results when using the clinical data only. As it can be seen, the *AUC* has reduced significantly when using the clinical data by itself. This is an indication that the EHG signals play significant roles in the classification process. The simulation results indicated that the *AUC* dropped noticeably with a best value achieved by the *POLYC* and *LOGLC* classifiers producing a value of 55% only.

**Table 9:** Classifier Performance for Clinical Data Only.

	Sensitivity	Specificity	AUC
Classifier	*Channel 3 0.34–1Hz Filter*	*Channel 3 0.34–1Hz Filter*	*Channel 3 0.34–1Hz Filter*
*LDC*	0.0000	1.0000	51%
*QDC*	1.0000	0.0384	51%
*UDC*	0.0000	0.9038	52%
*POLYC*	0.000	1.0000	55%
*LOGLC*	0.0000	1.0000	55%
*KNNC*	0.0000	0.9230	50%
*TREEC*	0.1428	0.8461	52%
*PARZENC*	0.0000	1.0000	49%
*SVC*	0.0000	1.0000	53%

The performance of *k-fold* cross-validation is compared with the results obtained from both the 80% *holdout* methods. Table 10 shows that the mean errors when using the clinical data only.

**Table 10:** Cross Validation Results for Clinical Data Only.

	80% Holdout: 30 Repetitions		Cross Val, 5 Folds, 1 Repetitions	Cross Val, 5 Folds, 6 Repetitions	
Classifiers	*Mean Err*	*SD*	*Mean Err*	*Mean Err*	*SD*
*LDC*	0.1354	0.0146	0.1399	0.1355	0.0053
*QDC*	0.8443	0.0338	0.8532	0.8559	0.0073
*UDC*	0.1953	0.0364	0.1930	0.1939	0.0062
*POLYC*	0.1278	0.0049	0.1300	0.1272	0.0013
*LOGLC*	0.1334	0.0139	0.1300	0.1322	0.0053
*KNNC*	0.1652	0.0289	0.1267	0.1283	0.0028
*TREEC*	0.2231	0.493	0.2126	0.2362	0.0227
*PARZENC*	0.1267	0.000	0.1267	0.1267	0.0000
*SVC*	0.1267	0.000	0.1267	0.1267	0.0000

Using the clinical data only, the *mean errors* and *k-fold* values are as expected and they are not considered statistically significant.

### Model Selection

**Figure 7:**
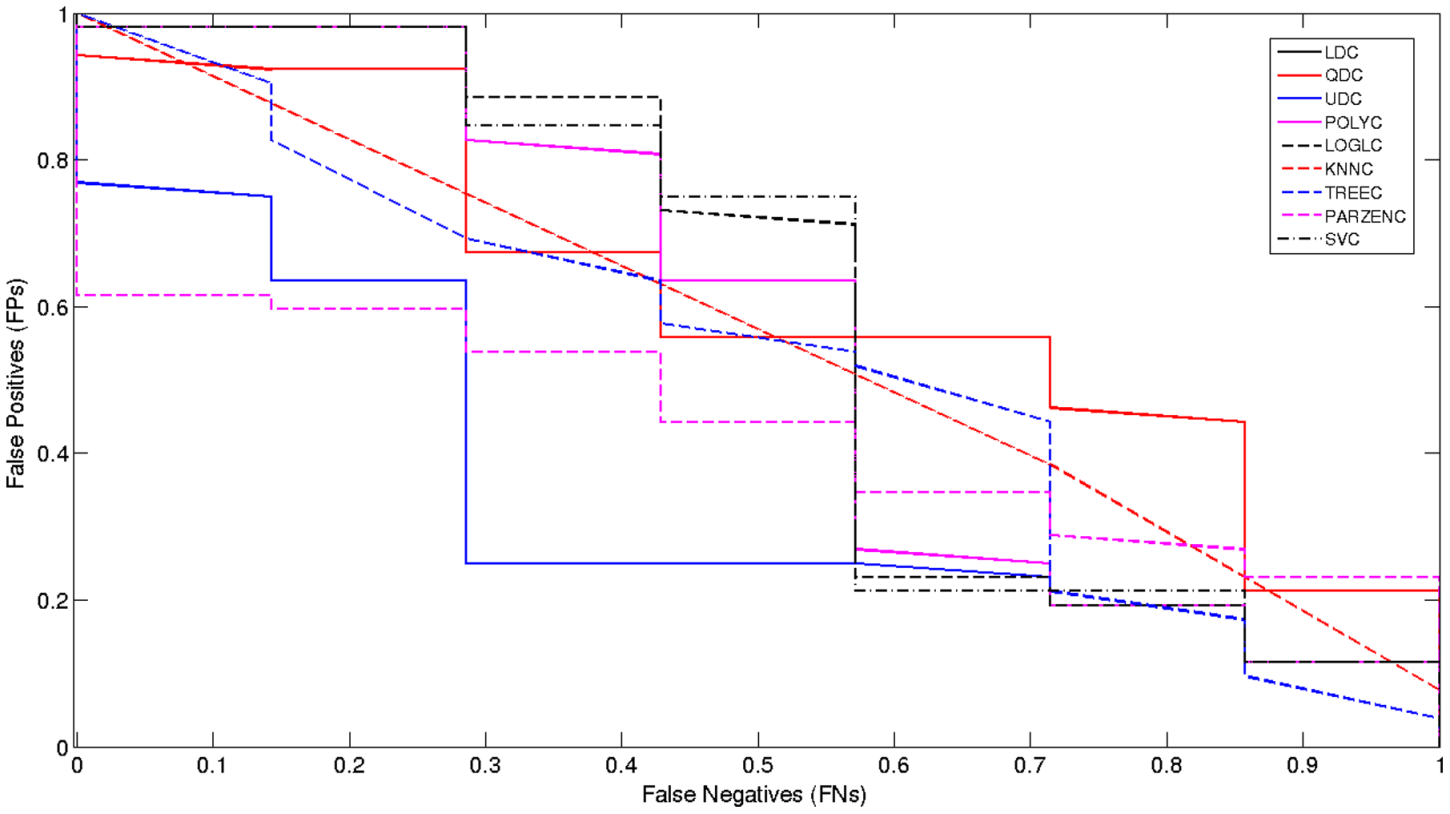
Received Operator Curve for Clinical Data Only.

Figure 7 shows that, when only using the clinical data, all classifiers have performed significantly worse than previous evaluations.

### Summary of Results

Table 11 and 12 illustrates a summary of the results for all four approaches. As it can be seen, the oversampled dataset, which utilized additional features, provided the best results with a significant increase in *sensitivity, specificity* and *AUC* values. In particular, using this method, *POLYC* has improved significantly.

**Table 11:** Summary of Classifier Performance for Original TPEHG Dataset and Oversampled Dataset Using SMOTE.

	Original TPEHG dataset			Oversampled using SMOTE		
	Sensitivity	Specificity	AUC	Sensitivity	Specificity	AUC
Classifier	*Channel* 3 0.34–1 Hz Filter	*Channel 3 0.34–1 Hz Filter*	*Channel 3 0.34–1 Hz Filter*	*Channel 3 0.34–1 Hz Filter*	*Channel 3 0.34–1 Hz Filter*	*Channel 3 0.34–1 Hz Filter*
*LDC*	0.0000	0.9807	53%	0.8653	0.8076	66%
*QDC*	0.0000	0.9807	53%	0.9230	0.8461	72%
*UDC*	0.0000	1.0000	52%	0.8269	0.8076	72%
*POLYC*	0.0000	0.9807	61%	0.8653	0.8076	86%
*LOGLC*	0.0000	0.9807	60%	0.8653	0.8269	86%
*KNNC*	0.0000	0.9230	53%	0.8653	0.8269	84%
*TREEC*	0.2857	0.8653	60%	0.9038	0.8269	89%
*PARZENC*	0.0000	1.0000	50%	0.5961	0.9615	72%
*SVC*	0.0000	1.0000	61%	0.8076	0.7692	78%

**Table 12:** Summary of Classifier Performance for Oversampling with Additional Features and Clinical Data Only.

	Oversampling with Additional Features			Clinical Data Only		
	Sensitivity	Specificity	AUC	Sensitivity	Specificity	AUC
Classifier	*Channel 3 0.34–1 Hz Filter*	*Channel 3 0.34–1 Hz Filter*	*Channel 3 0.34–1 Hz Filter*	*Channel 3 0.34–1 Hz Filter*	*Channel 3 0.34–1 Hz Filter*	*Channel 3 0.34–1 Hz Filter*
*LDC*	0.9666	0.9000	70%	0.0000	1.0000	51%
*QDC*	0.9666	0.1666	83%	1.0000	0.0384	51%
*UDC*	0.9666	0.1333	78%	0.0000	0.9038	52%
*POLYC*	0.9666	0.9000	95%	0.0000	1.0000	55%
*LOGLC*	0.9666	0.9000	94%	0.0000	1.0000	55%
*KNNC*	0.9333	0.8000	90%	0.0000	0.9230	50%
*TREEC*	0.9666	0.9000	93%	0.1428	0.8461	52%
*PARZENC*	0.9666	0.5666	59%	0.0000	1.0000	49%
*SVC*	0.9666	0.7000	92%	0.0000	1.0000	53%

The results illustrate that using machine learning techniques are encouraging. Within a wider context, this approach might be able to utilise real-life pregnancy data to predict, with high confidence, whether an expectant mother is likely to have a premature birth or proceed to full term.

## Discussion

Most studies, in the field of *EHG* classification, have focused on the diagnosis of *true* labour. This occurs at the stage when a woman believes, or suspects, she is in actual labour. This study has evaluated the use of a machine learning approach, using records from earlier stages of gestation, to predict *term* or *preterm* deliveries.

The initial classifications on the dataset (unbalanced) achieved a high *specificity*. However, this was at the cost of very poor *sensitivity*, below 20%. The *k-fold* cross-validation function was evaluated as a dataset splitting method to determine whether the *sensitivities* could be improved. However, the small improvements, in the *mean error*, were not statistically significant. The main problem occurred due to the disproportionate number of *term* records to *preterm* records. This causes bias in favour of true negatives or the majority class, as reported in [42]–[47]. The minimum error rate displayed across several of the classifiers, was 12.67%. This initially appeared to be a good error rate. However, the classifiers were simply classifying by minimising the probability of error, in the absence of sufficient evidence to help them to classify otherwise. It appeared as though most of the classifiers were classifying according to the prior probabilities of the classes, in order to minimise the error.

Using the SMOTE technique significantly improved the *sensitivity* and *specificity* rates, while maintaining high accuracy in the *AUC* values. The best classification algorithm was the *TREEC* classifier, which achieved 90% *sensitivity*, 83% *specificity*, and an *AUC* value of 89% with a 20% global error.

Using the oversampled clinical data the initial publication of the TPEHG dataset was in November 2010. However, in December 2012, clinical data became publically available. The final set of results shows that the overall performance of classifiers is improved further by including the information from the clinical dataset. Nonetheless, more recordings are needed, particularly more clinical information about the patients themselves. This would allow more reliable models to be constructed using the clinical and the EHG signals, which the findings in this paper support.

As it can be shown from Table 7, the Binary Decision Tree produced promising results of 93% accuracy, for the area under the curve, when the extra features are utilised in addition to the EHG signals. This is due to the feature of Binary decision trees, which use the engineering concept of divide and conquer. In this case, the binary decision tree will break down the complex decision-making problem into a collection of simpler decisions, thus providing a solution, which is often easier to interpret and understand. As indicated in Table 7, the best results have been achieved using the polynomial classifier. This is because the polynomial classifier adds polynomial features that can expand the input space, into higher dimensional space where linear seperability is possible.

While the results were very good, several issues were evident in the clinical data. Firstly, while the *weight* of the patient was provided, there was no information to say how tall they were, thus making it impossible to calculate their body mass index. Other features, such as *bleeding*, failed to show how often the *bleeding* occurred, or the amount of bleeding. In another example, the fact that someone *smoked* would be more informative if the number of cigarettes per day was provided. Nonetheless, while the data was vague, it was decided that the information might still be useful. The results suggest that the additional features further enhance the algorithms capability to separate *term* and *preterm* records.

From all the experiments performed, on the oversampled TPEHG dataset, with combined additional features, the *POLYC* classifier obtained the best result, as can be seen in Table 7. This classifier obtained 97% *sensitivity*, 90% *specificity*, a 95% *AUC* value and a global error of 8%. The *LOGLC,* and *TREEC* classifiers produced similar results, with overall *AUC* values of 94% and 93% respectively.

Generally, this paper produced significantly better results than those in [25], who report a *sensitivity* of 47%, *specificity* of 90%, and an overall error rate of 25%. Furthermore, the results have also been an improvement than those reported in [12]–[16], [54]–[58]. However, the findings in [31] produced a much lower error rate of 3.33%±1.3, a *sensitivity* rate of 100% and a *specificity* rate of 94%. Diab *et al.* have used several alternative techniques, including artificial neural networks and autoregressive models. However, it should be noted that the sample size is much smaller than the sample size in this paper (15 *preterm* and 15 *term*). The study in [31] also used a different data source, for their 30 records, compared to the TPEHG. Therefore, it is difficult, to make a direct comparison between that study and the study in this paper. Consequently, it is impossible to determine if the higher results are, in fact, better.

## Conclusions and Future Work

The rate of premature births has increased globally, which can lead to severe medical conditions and an increase in societal and economical costs. However, a better understanding of *preterm* births, and a strategic focus on prevention, is likely to improve health outcomes and reduce national healthcare service costs. A strong body of evidence has suggested that the analysis of uterine electrical signals from the abdominal surface (*EHG*) could provide a viable way of diagnosing true labour, and even predict *preterm* deliveries.

This paper utilises such *EHG* signals, within a supervised machine-learning paradigm, to classify *term* and *preterm* records. The focus of the paper has been to improve *sensitivity* rates, as it is more important to predict *preterm* delivery, as opposed to miss classifying a *term* pregnancy. As such, using the original TPEHG dataset, the number of *preterm* records (minority class) was considerably lower than the number of *term* records (majority class). Since the classifiers do not have enough *preterm* records to learn from, this led to the original results being quite poor. *AUC* values were no higher than 61% and, for the majority of the classifiers *sensitivity* was at 0%. In this instance, using the SMOTE technique, it has been necessary to oversample the *preterm* records. Oversampling the minority class enables the distribution between the two classes (*term* and *preterm*) to be more balanced. This technique significantly improved the results, with a maximum *AUC* value of 89% and *sensitivity* rate of 92%. Along with the SMOTE technique, as additional features became available this further improved the results. In this instance, a maximum *AUC* value of 95% and *sensitivities* of 97% were achieved. However, using only the clinical data produced significantly poorer results, with a maximum *AUC* value of 55% and the majority of *sensitivities* at 0%. As discussed, this could be due to the ambiguity of the clinical data. Nevertheless, these results are encouraging, and the approach shows an improvement on existing studies.

Despite these encouraging results, more in-depth research is still required. For example, regression analysis, using a larger number of classes, would be interesting. This would help to predict the expected delivery, in terms of the number of days or weeks, not just whether a woman is likely to deliver *term* or *preterm*.

Future work will evaluate different parameter adjustment settings. In addition, more advanced classification algorithms, and techniques, will be considered, including advanced Artificial Neural Network architectures, such as higher order and spiking neural networks. The investigation, and comparison, of features, such as fractal dimension and cepstrum analysis, autocorrelation zero crossing and correlation dimension, has also not been performed. Future work will investigate these techniques in a head-to-head comparison, with linear methods.

It would also be interesting to run a study in which the classification accuracy of features extracted, per-burst of *EMG*, are compared against those extracted from the whole record. In such a study, the same signals would be used. However, pre-processing would occur differently. According to the literature review, no such evaluation has been carried out. Future work will also combine signals from the various channels.

Overall, the study demonstrates that classification algorithms provide an interesting line of enquiry, when separating *term* and *preterm* delivery records.

## References

[1] WHO (2012) Born too soon: The Global Action Report on Preterm Birth.

[2] Baker PN, Kenny L (2011) Obstetrics by Ten Teachers. Hodder Arnold Press. 436 p.

[3] Greenough A (2012) Long Term Respiratory Outcomes of very Premature Birth (<32 weeks). Semin Fetal Neonatal Med 17(2): 73–76. 22300711 10.1016/j.siny.2012.01.009

[4] Mangham LJ, Petrou S, Doyle LW, Draper ES, Marlow N (2009) The Cost of Preterm Birth Throughout Childhood in England and Wales. Pediatrics 123(2): 312–327. 10.1542/peds.2008-1827 19171583

[5] Rattihalli R, Smith L, Field D (2012) Prevention of preterm births: are we looking in the wrong place? Archives of disease in childhood. Fetal and neonatal 97(3): 160–1. 10.1136/archdischild-2011-301096 22247417

[6] Goldenberg RL, Culhane JF, Iams JD, Romero R (2008) Epidemiology and causes of preterm birth. The Lancet 371(9606): 75–84. 10.1016/S0140-6736(08)60074-4 PMC7134569 18177778

[7] McPheeters M, Miller WC, Hartmann KE, Savitz DA, Kaufman JS, et al (2005) The Epidemiology of Threatened Premature Labor: A Prospective Cohort Study. American journal of obstetrics and gynaecology 192(4): 1325–9. 10.1016/j.ajog.2004.12.055 15846230

[8] Lucovnik M, Kuon RJ, Chambliss LR, Maner WL, Shi SQ, et al (2011) Use of uterine electromyography to diagnose term and preterm labor. Acta Obstetricia et Gynecologica Scandinavica 90(2): 150–157. 21241260 10.1111/j.1600-0412.2010.01031.x PMC3151256

[9] Muglia LJ, Katz M (2010) The Enigma of Spontaneous Preterm Birth. N Engl J Med 362(6): 529–35. 20147718 10.1056/NEJMra0904308

[10] Fele-Žorž G, Kavšek G, Novak-Antolič Z, Jager F (2008) A comparison of various linear and non-linear signal processing techniques to separate uterine EMG records of term and pre-term delivery groups. Medical & biological engineering & computing 46(9): 911–22. 18437439 10.1007/s11517-008-0350-y

[11] Doret M (2005) Uterine Electromyograpy Characteristics for early Diagnosis of Mifepristone-induced Preterm Labour. Obstetrics and Gynecology 105(4): 822–30. 15802412 10.1097/01.AOG.0000157110.62926.d7

[12] Moslem B, Khalil M, Diab MO, Chkeir A, Marque C (2011) A Multisensor Data Fusion Approach for Improving the Classification Accuracy of Uterine EMG Signals. 18^th^ IEEE International Conference on Electronics, Circuits and Systems (ICECS): 93–96.

[13] Moslem B, Khalil M, Diab MO, Marque C (2012) Classification of multichannel uterine EMG signals by using a weighted majority voting decision fusion rule. 16^th^ IEEE Mediterranean Electrotechnical Conference: 331–334.

[14] Moslem B, Khalil M, Diab M (2011) Combining multiple support vector machines for boosting the classification accuracy of uterine EMG signals. 18^th^ IEEE International Conference on Electronics, Circuits and Systems (ICECS): 631–634.

[15] Moslem B, Karlsson B, Diab MO, Khalil M, Marque C (2011) Classification Performance of the Frequency-Related Parameters Derived from Uterine EMG Signals. 33^rd^ Annual International Conference of the IEEE Engineering in Medicine and Biology Society: 3371–4. 10.1109/IEMBS.2011.6090913 22255062

[16] Moslem B, Diab MO, Khalil M, Marque C (2011) Classification of multichannel uterine EMG signals by using unsupervised competitive learning. IEEE Workshop on Signal Processing Systems: 267–272.

[17] Moslem B, Diab MO, Marque C, Khalil M (2011) Classification of multichannel Uterine EMG Signals. 33^rd^ Annual International Conference of the IEEE Engineering in Medicine and Biology Society: 2602–5. 10.1109/IEMBS.2011.6090718 22254874

[18] Rabotti C, Mischi M, Oei SG, Bergmans JWM (2010) Noninvasive estimation of the electrohysterographic action-potential conduction velocity. IEEE transactions on bio-medical engineering 57(9): 2178–87. 20460202 10.1109/TBME.2010.2049111

[19] Buhimschi C, Boyle MB, Garfield RE (1997) Electrical activity of the human uterus during pregnancy as recorded from the abdominal surface. Obstetrics & Gynecology 90(1): 102–111. 9207823 10.1016/S0029-7844(97)83837-9

[20] Lammers WJ (2013) The Electrical Activities of the Uterus During Pregnancy. Reproductive Sciences 20(2): 182–9. 22649122 10.1177/1933719112446082

[21] Garfield RE, Maner WL (2007) Physiology and Electrical Activity of Uterine Contractions. Seminars in Cell and Developmental Biology 18(3): 289–95. 17659954 10.1016/j.semcdb.2007.05.004 PMC2048588

[22] Gondry J, Marque C, Duchene J, Cabrol D (1993) Electrohysterography during Pregnancy: Preliminary Report. Biomedical Instrumentation and Technology/Association for the Advancement of Medical Instrumentation 27(4): 318–324. 8369867

[23] Lucovnik M, Maner WL, Chambliss LR, Blumrick R, Balducci J, et al (2011) Noninvasive uterine electromyography for prediction of preterm delivery. American journal of obstetrics and gynecology 204(3): 228.e1–10. 21145033 10.1016/j.ajog.2010.09.024 PMC3090039

[24] Leman H, Marque C, Gondry J (1999) Use of the electrohysterogram signal for characterization of contractions during pregnancy. IEEE transactions on bio-medical engineering 46(10): 1222–9. 10513127 10.1109/10.790499

[25] Verdenik I, Pajntar M, Leskosek B (2001) Uterine electrical activity as predictor of preterm birth in women with preterm contractions. European journal of obstetrics, gynecology, and reproductive biology 95(2): 149–53. 10.1016/s0301-2115(00)00418-8 11301159

[26] Maner WL, Garfield RE, Maul H, Olson G, Saade G (2003) Predicting term and preterm delivery with transabdominal uterine electromyography. Obstetrics & Gynecology 101(6): 1254–1260. 12798533 10.1016/s0029-7844(03)00341-7

[27] Marque CK, Terrien J, Rihana S, Germain G (2007) Preterm labour detection by use of a biophysical marker: the uterine electrical activity. BMC pregnancy and childbirth 7(Suppl 1): S5. 10.1186/1471-2393-7-S1-S5 PMC1892062 17570165

[28] Maner WL, Garfield RE (2007) Identification of human term and preterm labor using artificial neural networks on uterine electromyography data. Annals of biomedical engineering 35(3): 465–73. 17226089 10.1007/s10439-006-9248-8

[29] Hassan M, Terrien J, Marque C, Karlsson B (2011) Comparison between Approximate Entropy, Correntropy and Time Reversibility: Application to Uterine Electromyogram Signals. Medical engineering & physics 33(8): 980–6. 21497127 10.1016/j.medengphy.2011.03.010

[30] Buhimschi C, Boyle MB, Saade GR, Garfield RE (1998) Uterine activity during pregnancy and labor assessed by simultaneous recordings from the myometrium and abdominal surface in the rat. American journal of obstetrics and gynecology 178(4): 811–22. 9579450 10.1016/s0002-9378(98)70498-3

[31] Diab MO, El-Merhie A, El-Halabi N, Khoder L (2010) Classification of Uterine EMG signals using Supervised Classification method. Biomedical Science and Engineering 3(9): 837–842.

[32] Carre P, Leman H, Fernandez C, Marque C (1998) Denoising of the Uterine EHG by an Undecimated Wavelet Transform. IEEE transactions on bio-medical engineering 45(9): 1104–13. 9735560 10.1109/10.709554

[33] Maner WL, MacKay LB, Saade GR, Garfield RE (2006) Characterization of abdominally acquired uterine electrical signals in humans, using a non-linear analytic method. Medical & biological engineering & computing 44(1–2): 117–23. 16929929 10.1007/s11517-005-0011-3

[34] Vinken MP, Rabotti C, Mischi M, Oei SG (2009) Accuracy of frequency-related parameters of the electrohysterogram for predicting preterm delivery. Obstetrical & gynecological survey 64(8): 529. 19624864 10.1097/OGX.0b013e3181a8c6b1

[35] Garfiled RE, Maner WL, Maul H, Saade GR (2005) Use of Uterine EMG and cervical LIF in Monitoring Pregnant Patients. International Journal of Obstetrics & Gynaecology 112: 103–8. 10.1111/j.1471-0528.2005.00596.x 15715606

[36] Buhimschi C, Garfield RE (1996) Uterine contractility as assessed by abdominal surface recording of electromyographic activity in rats during pregnancy. American journal of obstetrics and gynecology 174(2): 744–53. 8623816 10.1016/s0002-9378(96)70459-3

[37] Richman JS, Moorman JR (2000) Physiological time-series analysis using approximate entropy and sample entropy. American Journal of Physiology – Heart and Circulatory Physiology 278(6): H2039–49. 10843903 10.1152/ajpheart.2000.278.6.H2039

[38] Charniak E (1991) Bayesian Networks without Tears. AI Magazine 12(4): 50–63.

[39] Baghamoradi S, Naji M, Aryadoost H (2011) Evaluation of cepstral analysis of EHG signals to prediction of preterm labor. 18^th^ Iranian Conference on Biomedical Engineering: 1–3.

[40] Diab MO, Marque C, Khalil MA (2007) Classification for Uterine EMG Signals/: Comparison between AR Model and Statistical Classification Method. International Journal of Computational Cognition 5(1): 8–14.

[41] Chawla NV, Bowyer KW, Hall LO, Kegelmeyer WP (2002) SMOTE: Synthetic Minority Over-Sampling Technique. Journal of Artificial Intelligence Research 16(1): 321–357.

[42] Taft LM, Evans RS, Shyu CR, Egger MJ, Chawla N, et al. Countering imbalanced datasets to improve adverse drug event predictive models in labor and delivery. Journal of Biomedical Informatics 42(2): 356–364. 18824133 10.1016/j.jbi.2008.09.001 PMC2692750

[43] Sun T, Zhang R, Wang J, Li X, Guo X (2013) Computer-Aided Diagnosis for Early-Stage Lung Cancer Based on Longitudinal and Balanced Data. PLOS One 8(5): e63559. 23691066 10.1371/journal.pone.0063559 PMC3655169

[44] Lin W, Chen JJ (2013) Class-imbalanced classifiers for high-dimensional data. Briefings in Bioinformatics 14(1): 13–26. 22408190 10.1093/bib/bbs006

[45] Nahar J, Imam T, Tickle KS, Ali ABMS, Chen YP (2012) Computational Intelligence for Microarray Data and Biomedical Image Analysis for the Early Diagnosis of Breast Cancer. Expert Systems with Applications 39(16): 12371–12377.

[46] Blagus R, Lusa L (2013) SMOTE for High-Dimensional Class-Imbalanced Data. BMC Bioinformatics 14(106): 1–16. 23522326 10.1186/1471-2105-14-106 PMC3648438

[47] Wang Y, Simon M, Bonde P, Harris BU, Teuteberg JJ, et al (2012) Prognosis of Right Ventricular Failure in Patients with Left Ventricular Assist Device Based on Decision Tree with SMOTE. Transactions on Information Technology in Biomedicine 16(3): 383–90. 22334033 10.1109/TITB.2012.2187458

[48] van der Heijde F, Duin RPW, de Ridder D, Tax DMJ (2005) Classification, Parameter Estimation and State Estimation. Wiley-Blackwell. 440 p.

[49] Russell S, Norvig P (2010) Artificial Intelligence – A Modern Approach. Prentice Hall. 1152 p.

[50] Salkind NJ (2008) Statistics for people who (think they) hate statistics. Sage Publications. 424 p.

[51] Fawcett T (2006) An Introduction to ROC analysis. Pattern Recognition Letters 27(8): 861–874.

[52] Lasko TA, Bhagwat JG, Zou KH, Ohno-Machada L (2005) The use of receiver operating characteristic curves in biomedical informatics. Journal of biomedical informatics 38(5): 404–15. 16198999 10.1016/j.jbi.2005.02.008

[53] Tong L, Change Y, Lin S (2011) Determining the optimal re-sampling strategy for a classification model with imbalanced data using design of experiments and response surface methodologies. Expert Systems with Applications 38(4): 4222–4227.

[54] Moslem B, Diab MO, Marque C, Khalil M (2011) Classification of multichannel uterine EMG signals. IEEE Annual International Conference on Engineering in Medicine and Biology Society: 2602–5. 10.1109/IEMBS.2011.6090718 22254874

[55] Diab MO, Moslem B, Khalil M, Marque C (2012) Classification of Uterine EMG Signals by using Normalized Wavelet Packet Energy. 16^th^ IEEE Mediterranean Electrotechnical Conference: 335–338.

[56] Moslem B, Diab MO, Khalil M, Marque C (2012) Classification of Multichannel Uterine EMG Signals Using a Reduced Number of Channels. 8^th^ International Symposium on Mechatronics and its Applications: 1–4.

[57] Hassan M, Alexandersson A, Terrien J, Muszynski C, Marque C, et al.. (2012) Better Pregnancy Monitoring using Nonlinear Correlation Analysis of External Uterine Electromyography. IEEE transactions on bio-medical engineering 60(4) 1160–1166. 10.1109/TBME.2012.2229279 23192483

[58] Diab A, Hassan M, Marque C, Karlsson B (2013) Quantitative Performance Analysis of Four Methods of Evaluating Signal Nonlinearity: Application to Uterine EMG Signals. IEEE Engineering in Medicine and Biology Society: 1045–1048. 10.1109/EMBC.2012.6346113 23366074

